# Supplementary material for: Correlation of Myeloid-Derived Suppressor Cell Expansion with Upregulated Transposable Elements in Severe COVID-19 Unveiled in Single-Cell RNA Sequencing Reanalysis
Source: Biomedicines. 2024 Jan 29;12(2):315. doi: 10.3390/biomedicines12020315 (PMC10887269; doi:10.3390/biomedicines12020315)
Supplement: Supplementary file 1 [file biomedicines-12-00315-s001.zip › biomedicines-2787101-supplementary.pdf]

Correlation of myeloid-derived suppressor cell expansion with upregulated transposable elements in severe COVID-19 unveiled in single-cell RNA sequencing reanalysis

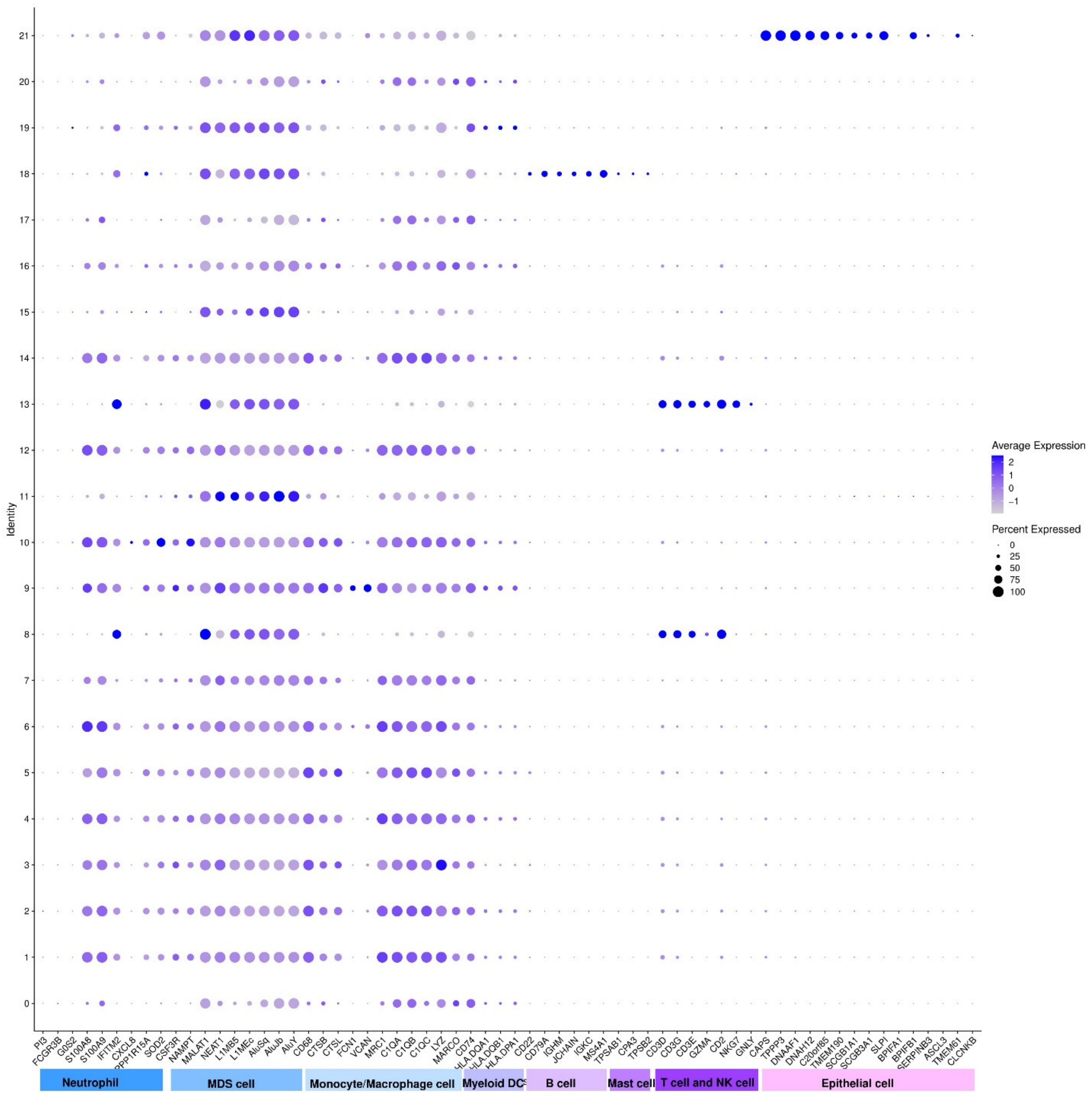

Fig. S1. Dot plot of canonical marker gene expression in the healthy population.

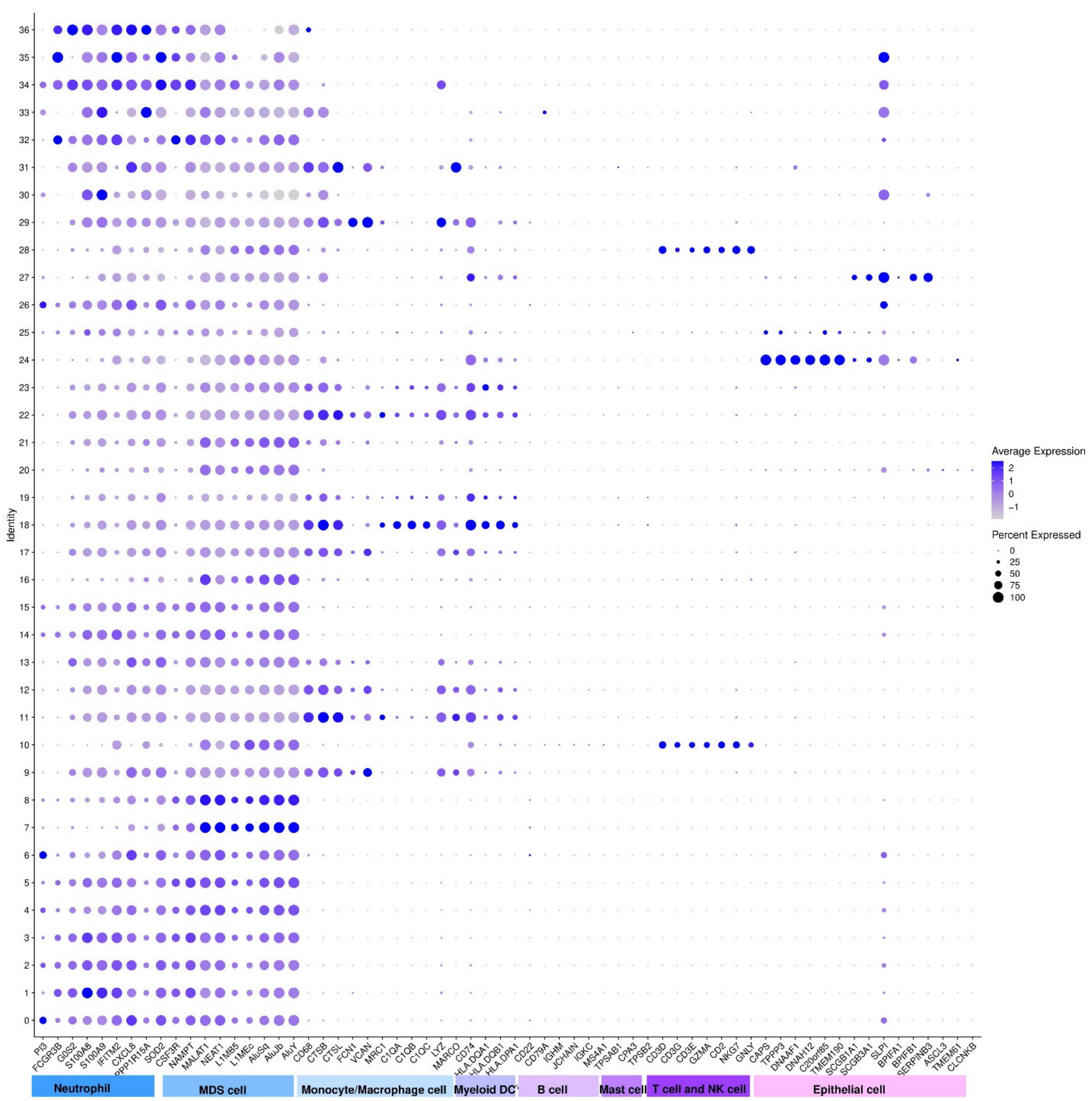

Fig. S2. Dot plot of canonical marker gene expression in alive population.

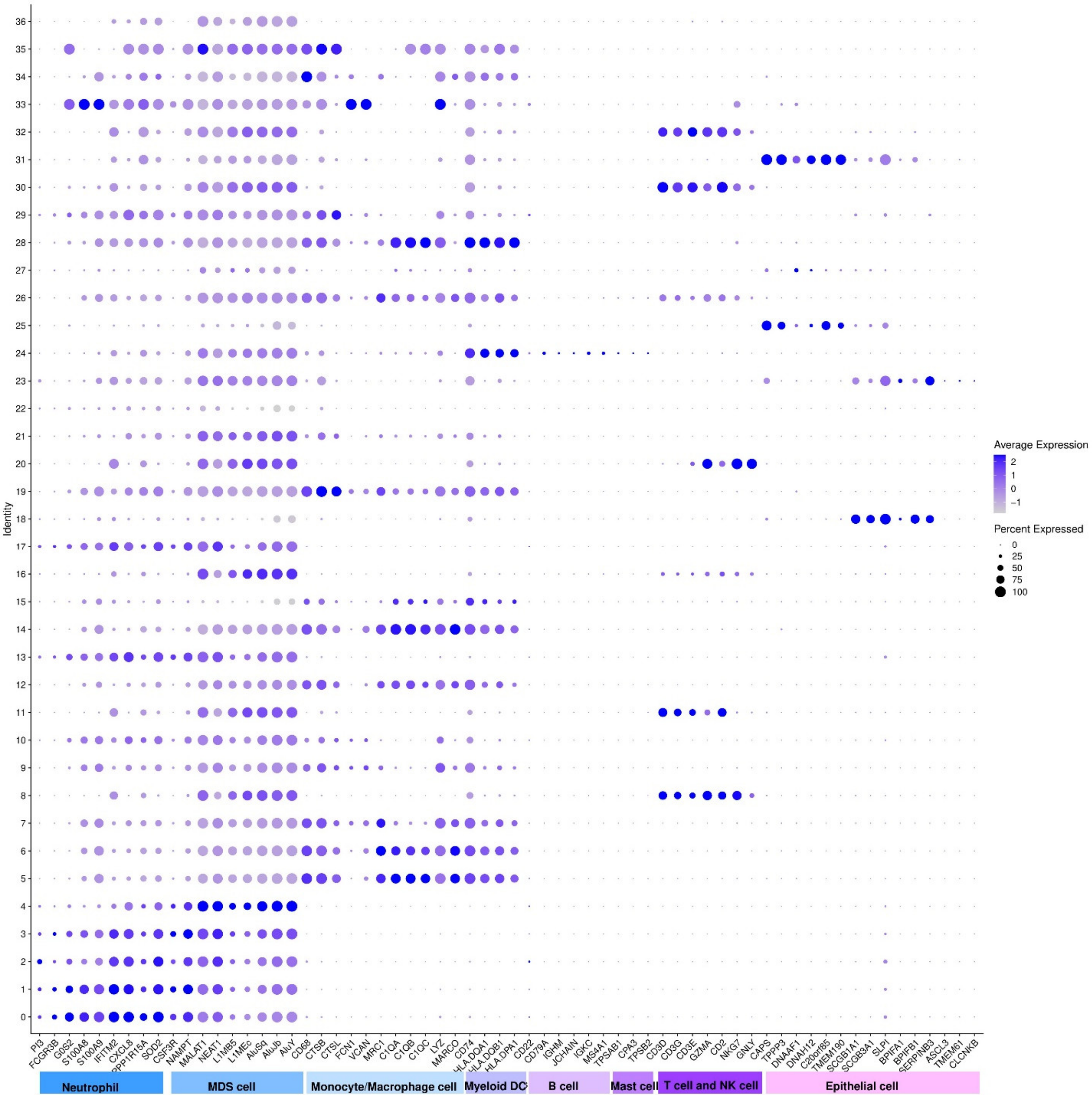

Fig. S3. Dot plot of canonical marker gene expression in dead population.

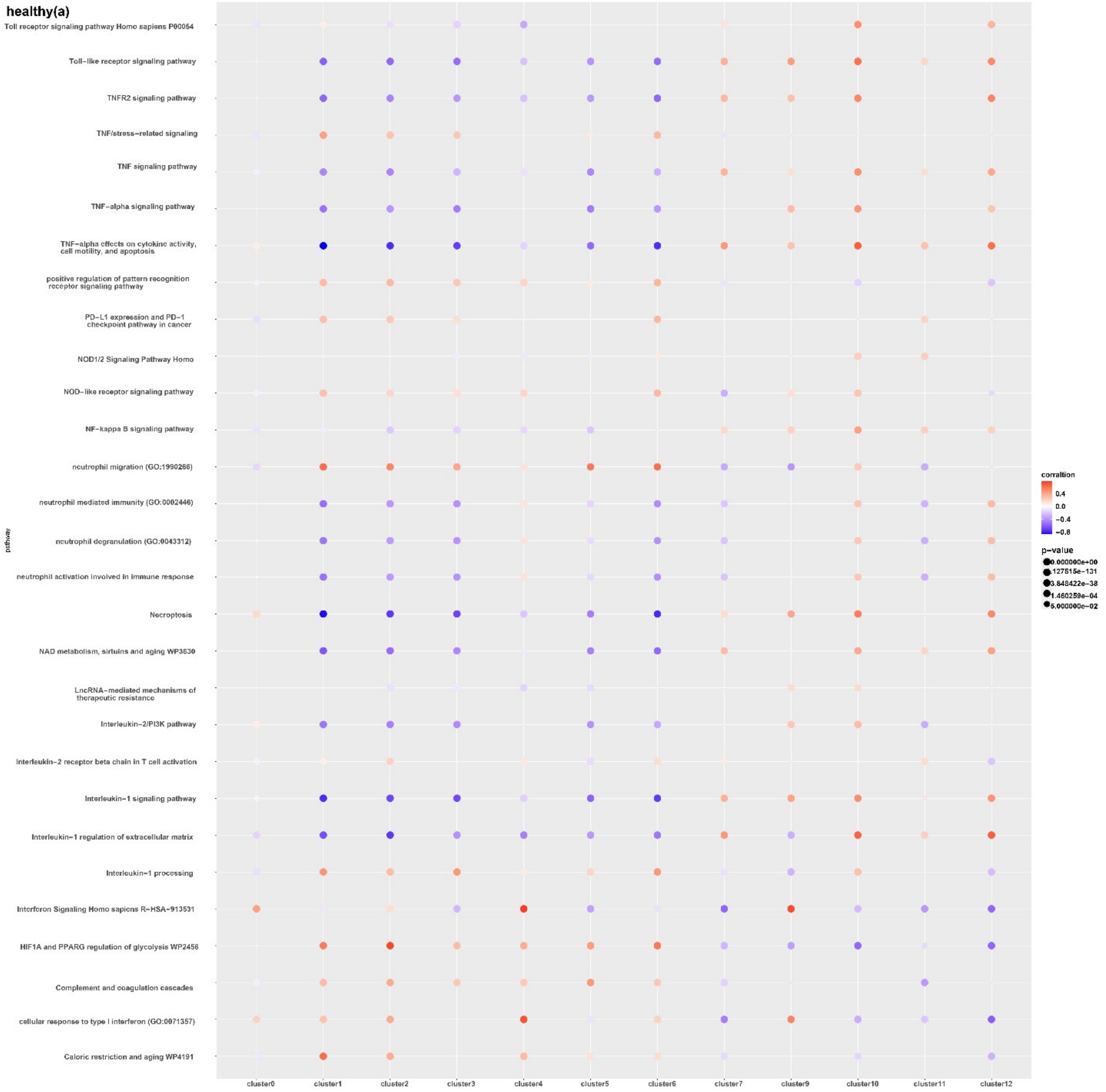

Fig.S4. Correlation between the selected 29 pathways and the overexpressed genes in myeloid cell type clusters of healthy population.

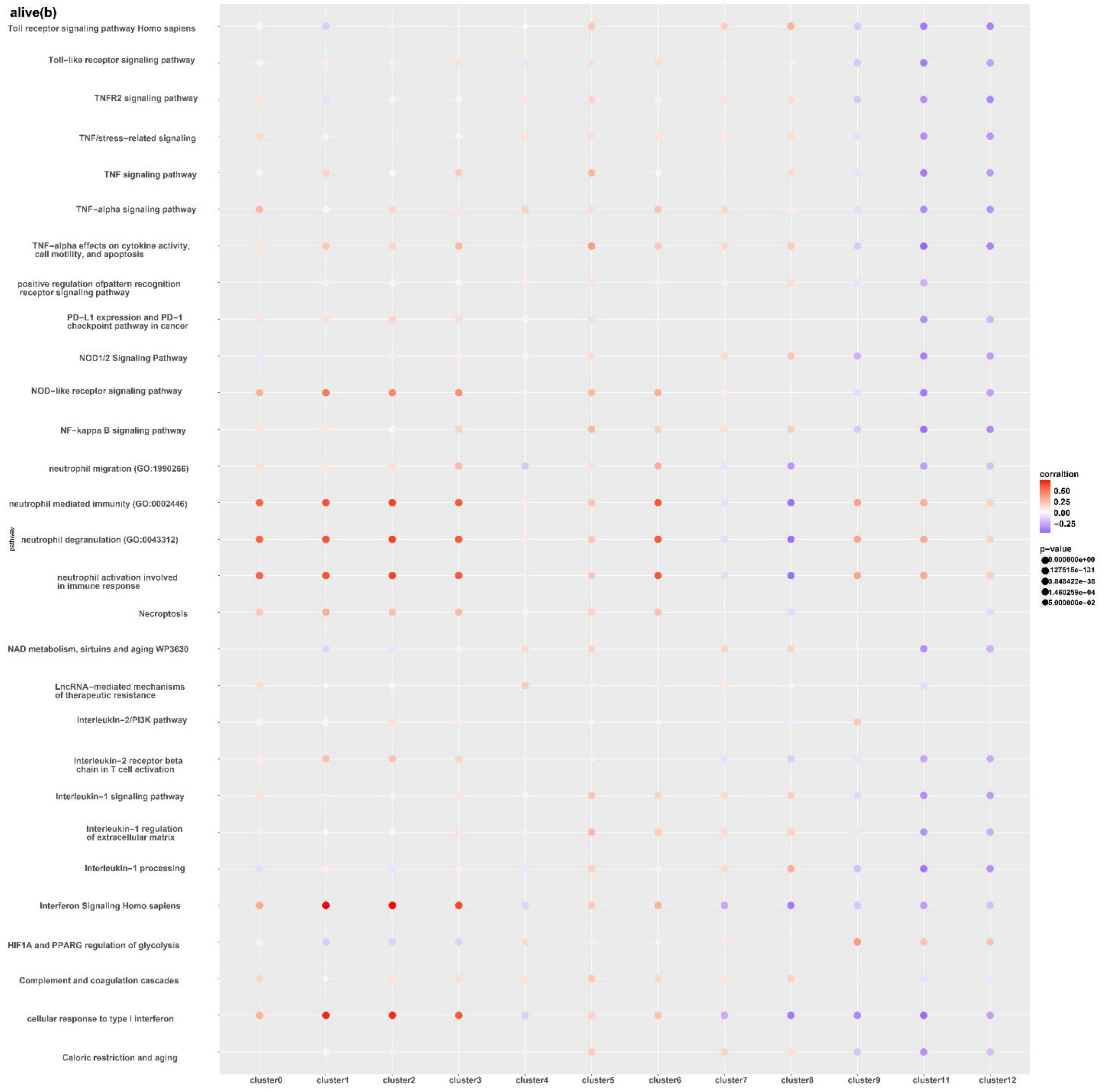

Fig.S5. Correlation between the selected 29 pathways and the overexpressed genes in myeloid cell type clusters of alive population.

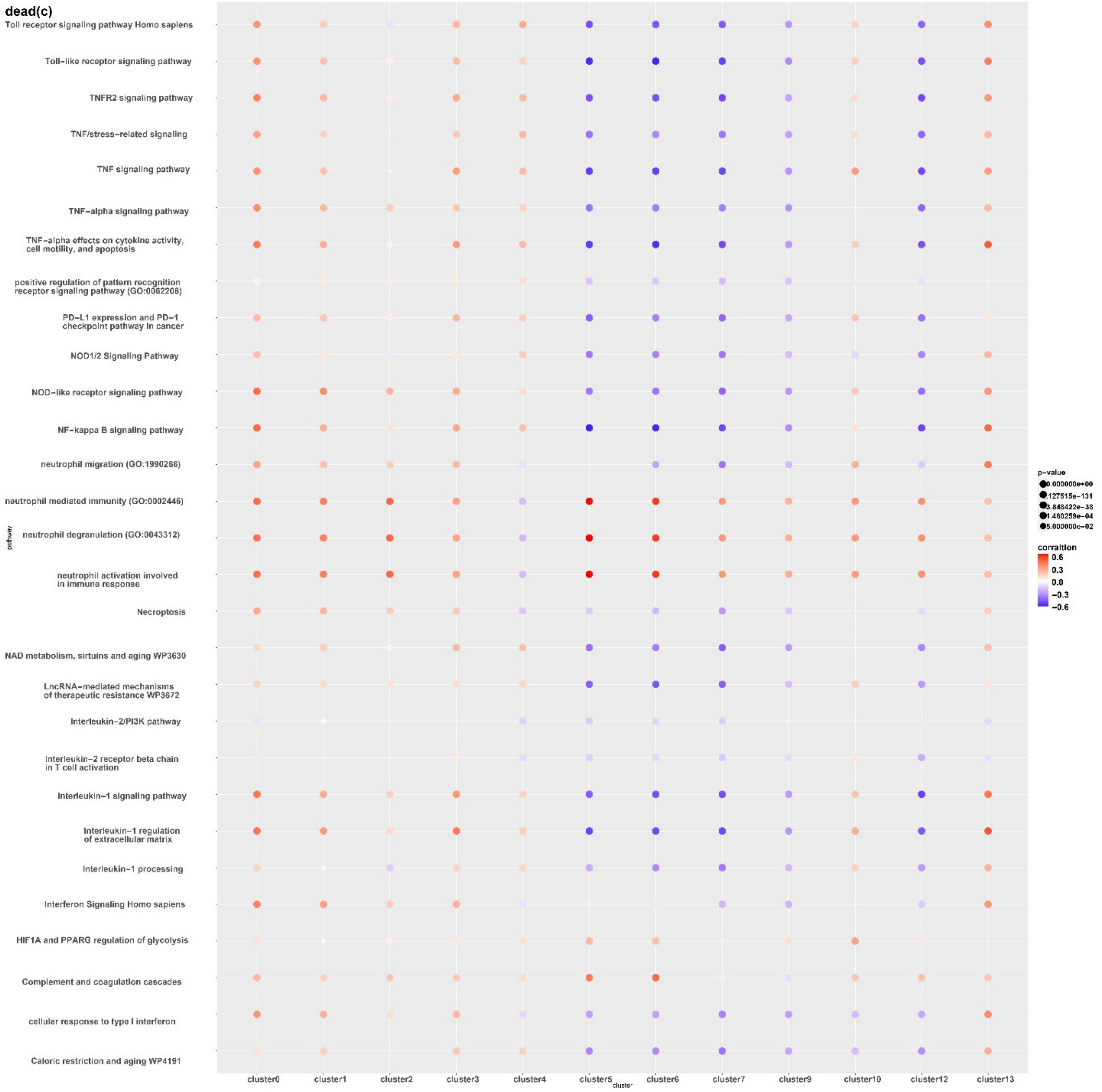

Fig.S6. Correlation between the selected 29 pathways and the overexpressed genes in myeloid cell type1 clusters of dead population.
